# Epigenetics in Alzheimer’s disease: from molecular mechanisms to emerging novel biomarkers

**DOI:** 10.3389/fnagi.2026.1959361

**Published:** 2026-10-05

**Authors:** Elisabetta D’Aversa, Miriana Grisafi, Salvatore Pernagallo, Francesca Salvatori, Ajay V. Singh, Alessia Nucciarone, Veronica Tisato, Donato Gemmati

**Affiliations:** 1 Department of Translational Medicine, University of Ferrara, Ferrara, Italy; 2 Center for Hemostasis and Thrombosis, University of Ferrara, Ferrara, Italy; 3 Department of Environmental Sciences and Prevention, University of Ferrara, Ferrara, Italy; 4 Department of Chemical and Product Safety, German Federal Institute for Risk Assessment (BfR), Berlin, Germany; 5 University Strategic Centre for Studies on Gender Medicine, University of Ferrara, Ferrara, Italy; 6 LTTA Center, University of Ferrara, Ferrara, Italy

**Keywords:** Alzheimer’s disease, epigenetics, methylation, histone marks, mitoepigenetics, non-coding RNAs, biomarkers

## Abstract

Alzheimer’s disease (AD) is an age-related progressive neurodegenerative disorder that represents the leading cause of dementia worldwide, affecting over 50 million people. This global burden is expected to rise dramatically over the coming decades because of global population aging, representing a public health concern. Despite significant advances in understanding AD pathophysiology, early diagnosis remains challenging, emphasizing the urgent need for novel, reliable diagnostic and predictive biomarkers capable of identifying individuals at risk of progression during the preclinical stages of the disease. Growing evidence indicates that epigenetics constitutes a dynamic interface through which aging and environmental exposures influence gene expression and cellular homeostasis, thereby contributing to AD susceptibility and progression. The present review summarizes current knowledge on the epigenetic landscape of AD, from canonical epigenetic mechanisms, including aberrant DNA methylation, histone modifications, and non-coding RNAs (ncRNAs), to the emerging role of mitoepigenetics and RNA methylation in disease-associated molecular mechanisms. Finally, we discuss the potential of epigenetic alterations as promising innovative biomarkers of AD, providing insights into novel avenues for diagnostic and therapeutic intervention.

## 1 Introduction

Alzheimer’s disease (AD) is an age-related neurodegenerative disorder clinically characterized by progressive cognitive decline, including memory deterioration and impaired thinking, and changes in behavior, leading to a severe loss of independence and significantly impairing patients’ quality of life (Kamatham et al., 2024; Paniri et al., 2024; Tenchov et al., 2024). The progressive increase in life expectancy has led to a dramatic rise in the global prevalence of AD, with profound social, economic, and healthcare implications, representing one of the most pressing healthcare challenges of the twenty-first century (Gianfredi et al., 2025). The main pathological hallmarks of AD (Figure 1) include the progressive abnormal accumulation of amyloid beta (Aβ) plaques and neurofibrillary tangles made of hyperphosphorylated tau protein in the brain. These pathological features interfere with neuronal communication, leading to synaptic dysfunction, and contribute to activate inflammatory cascades, ultimately resulting in neuronal damage (Ahmadi et al., 2025). Despite significant advances in understanding AD pathophysiology, the molecular bases underlying disease onset and progression, as well as the exact cause-and-effect relationship, remain not fully understood, highlighting the remarkable biological complexity of the disease (Kumari et al., 2025).

**FIGURE 1:**
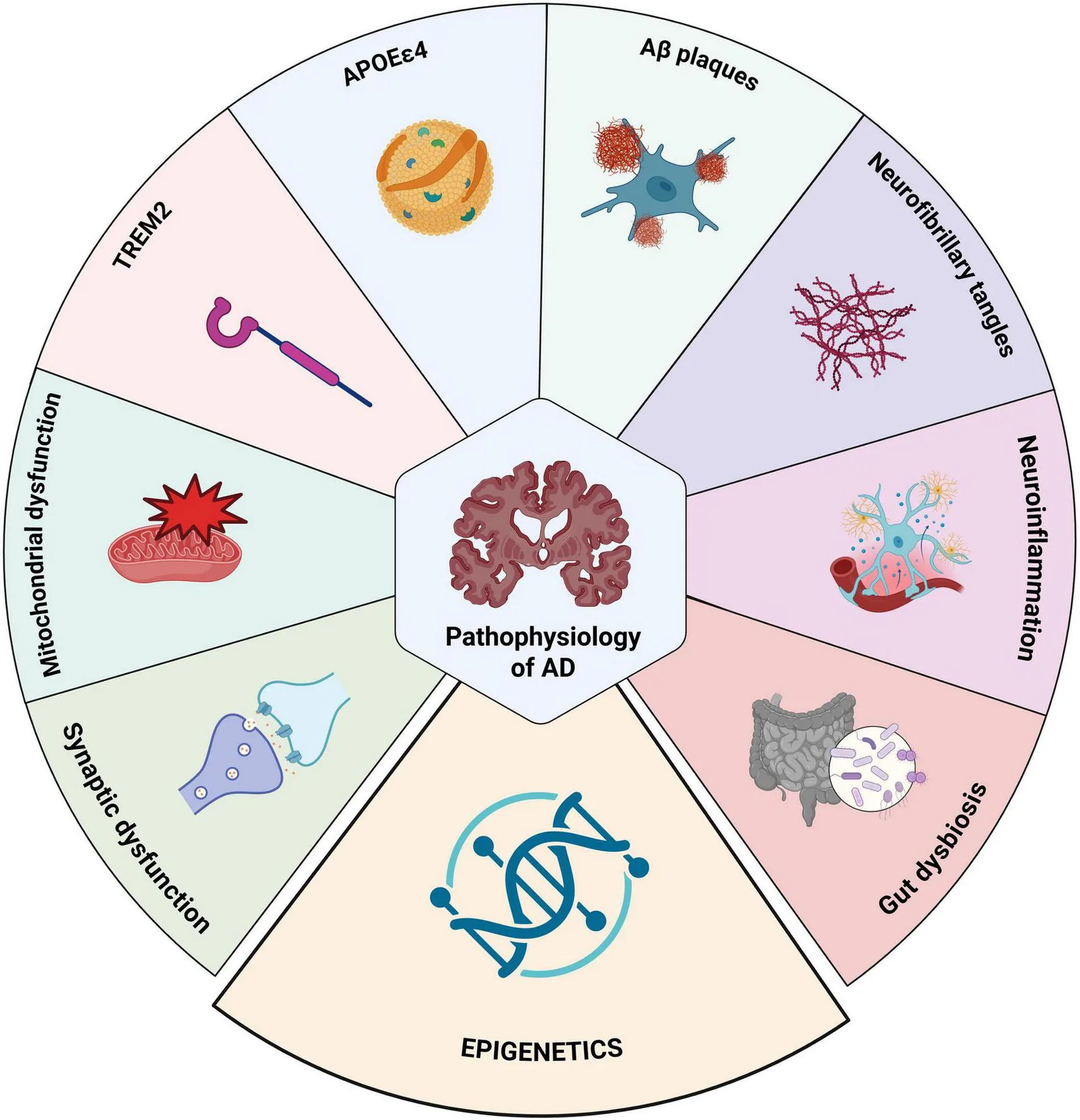
Epigenetics as central regulator of AD pathological features. The schematic illustrates the multifactorial nature of AD, highlighting the key molecular and cellular processes involved in disease onset and progression, including Aβ plaque accumulation, neurofibrillary tangle formation, neuroinflammation, gut dysbiosis, synaptic dysfunction, mitochondrial dysfunction, and genetic risk factors such as *APOE*ε*4* and *TREM2*. Epigenetics act as dynamic modulator connecting susceptibility with environmental influences and age-driven alterations, thereby orchestrating gene expression changes that contribute to AD pathogenesis.

AD is recognized as a multifactorial disorder resulting from an intricate interplay between genetics, environmental exposures and lifestyle-related factors (Ahmadi et al., 2025; Mertaş and Boşgelmez, 2025). These factors do not act independently but may interact through gene–environment (G × E) interactions, whereby environmental and lifestyle exposures can modify the effects of genetic variants on disease susceptibility and progression (Eid et al., 2019). While familial forms of AD (FAD) are associated with mutations in genes (e.g., *APP*, *PSEN1*, *PSEN2*) that promote Aβ plaque formation, the majority of cases are sporadic and involve multiple factors. Among all, *APOE*ε*4* allele and, more recently recognized, gene variants in *TREM2*, both involved in regulation of microglial inflammatory responses, amyloid clearance, and tau pathology, represent the strongest genetic risk factors for AD (Tisato et al., 2018; Liu A. et al., 2025; Qi et al., 2025), together with female sex, a well-established AD independent risk factor (Price et al., 2025). Additional contributing mechanisms are mitochondrial dysfunction and the related oxidative stress, which further exacerbate neuronal vulnerability by impairing energy metabolism, increasing reactive oxygen species production, and promoting apoptotic pathways (Tisato et al., 2016; Cervellati et al., 2019; Misrani et al., 2021; D’Alessandro et al., 2025). More recently, gut microbiota dysbiosis has emerged as an important contributor to AD pathogenesis altering the brain homeostasis through the microbiota–gut–brain axis leading to neuroinflammation and exacerbating Aβ deposition, tau pathology, and neuronal dysfunction (Lei et al., 2025; Ohara and Hsiao, 2025).

Collectively, these findings underscore the multifactorial nature of AD, which results from the dynamic interplay of several pathogenic mechanisms. Although these mechanisms have been extensively characterized, the regulatory framework that coordinates their interaction during disease onset and progression remains one of the major challenges in AD research. Emerging evidence highlights the crucial role of epigenetics as the integrative layer that fulfills this role, linking susceptibility and environmental exposure (Figure 1; Villa and Combi, 2024; Hu et al., 2025; Wang et al., 2025). Epigenetic modifications are dynamic and potentially reversible processes that regulate gene expression without altering the DNA sequence (Gemmati et al., 2025b; Tisato et al., 2025). These mechanisms include DNA and RNA methylation, histone modifications, and non-coding RNAs (ncRNAs), all of which play key roles in neuronal development, synaptic plasticity, memory formation, and neuroinflammatory responses (Campbell and Wood, 2019; Villa and Combi, 2024). Furthermore, aging–the major non-modifiable risk factor for AD–is associated with profound epigenomic remodeling, supporting the hypothesis that age-related epigenetic alterations, reflected by changes in epigenetic clocks, may promote neurodegeneration (Brombo et al., 2018; Liu Y. et al., 2024; Zhao et al., 2025). Moreover, epigenetic signatures are highly influenced by environmental and lifestyle factors (Klibaner-Schiff et al., 2024). The reversible nature of epigenetic modifications makes them attractive candidates as promising biomarkers not only for early diagnosis and disease progression but also as potential therapeutic targets of considerable interest (Wisman et al., 2024). Therefore, a comprehensive understanding of epigenetic dysregulation in AD may provide new opportunities for precision medicine approaches.

From this perspective, this review provides a comprehensive overview of the current knowledge of epigenetic mechanisms in AD. We discuss how alterations in DNA and RNA methylation, histone modifications, mitochondrial epigenetics and ncRNAs drive AD pathogenesis and progression, provide a deeper understanding of their roles in the disease molecular complexity, and bring forth valuable insights in the development of novel diagnostic tools and targeted therapies, ultimately improving the management of AD.

## 2 Epigenetic mechanisms of AD

The epigenetic landscape of AD is highly complex and involves multiple interconnected mechanisms. These include genomic instability associated with aging, alterations in DNA methylation patterns, a wide range of histone modifications, dysregulation of ncRNAs, and epigenetic changes affecting mitochondrial DNA (mtDNA). As illustrated in Figure 2, this chapter provides an overview of the principal epigenetic mechanisms involved in AD pathogenesis, highlighting their molecular basis and contribution to neurodegeneration.

**FIGURE 2:**
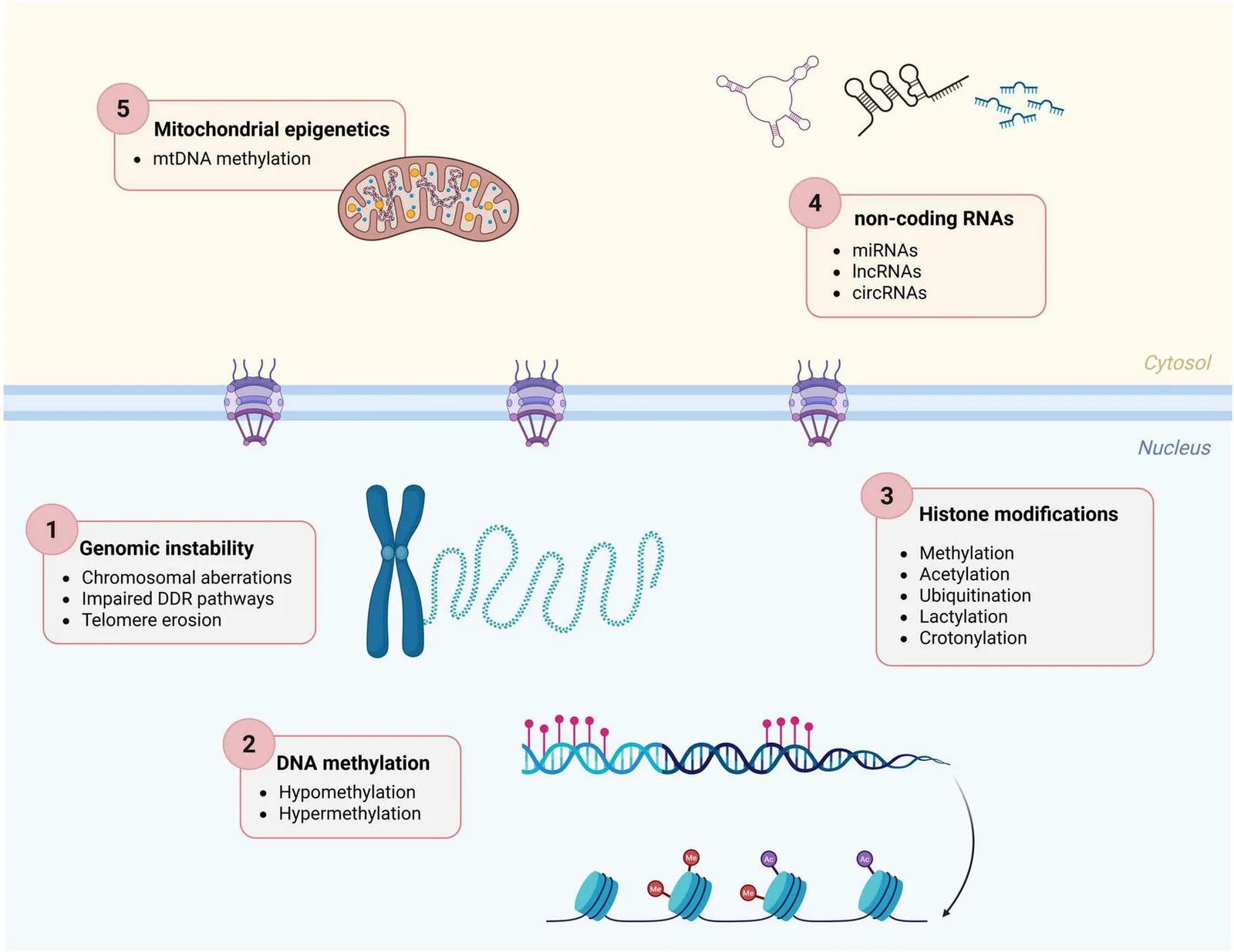
Overview of the principal epigenetic mechanisms involved in AD pathogenesis. (1) Genomic instability characterized by chromosomal aberrations, impaired DNA damage-repair (DDR) pathways and telomere erosion that accumulate with aging; (2) DNA hypomethylation and hypermethylation; (3) Histone modifications, such as methylation, acetylation, ubiquitination, lactylation, and crotonylation; (4) non-coding RNAs, including micro RNAs (miRNAs), long non-coding RNAs (lncRNAs), and circular RNAs (circRNAs); (5) Mitochondrial epigenetics, encompassing mitochondrial DNA (mtDNA) methylation.

### 2.1 Genomic instability

Genomic instability, including chromosomal aberrations and progressive telomere shortening (Figure 2), is not considered a bona fide epigenetic modification, but epigenetics and genomic instability are tightly connected in a bidirectional interplay, particularly relevant in age-related disorders, including AD (López-Gil et al., 2023). Specifically, epigenetic alterations, accumulated over the course of lifespan, influence chromatin organization, DNA repair efficacy, and telomere maintenance, thereby promoting genomic instability (Gemmati et al., 2025a). In turn, DNA damage and telomere attrition reshape the epigenetic landscape, establishing a self-reinforcing cycle that contributes to cellular senescence and neuronal vulnerability, and represents an early event in the pathogenesis of AD (Feng and Riddle, 2020; Soto-Palma et al., 2022; Zhao and Huai, 2023). AD brain displays increased DNA damage, particularly DNA double-strand breaks (DSBs), together with impaired DNA damage-repair (DDR) pathways, compared to a healthy aging brain (Thadathil et al., 2021). Persistent DNA damage accumulation promotes genomic rearrangements and disruption of three-dimensional genome organization, ultimately affecting transcriptional homeostasis and cellular functions (Thadathil et al., 2021; Dileep et al., 2023). Aberrant activation of DDR pathways can induce neuronal senescence-like phenotypes characterized by altered gene expression (Wong and Chow, 2023; Hughes et al., 2026). Several factors associated with AD, including oxidative stress, mitochondrial dysfunction, neuroinflammation, and Aβ/tau accumulation, may contribute to genomic instability by increasing genotoxic stress (Hou et al., 2017). Likewise, progressive telomere shortening and loss of telomere integrity can activate persistent DNA damage signaling, thereby exacerbating cellular senescence and inflammatory responses (Huang X. et al., 2025). A study by Crocco et al. (2023) demonstrated that patients with AD exhibit significantly shorter leukocyte telomere length compared with age-matched cognitively healthy controls, suggesting accelerated biological aging and impaired genome maintenance in AD. However, the use of telomere length as a marker of biological aging remains questioned. Telomere length is influenced by several biological and environmental factors, including chronological age, cell type and tissue, sex, genetic background, oxidative stress, and lifestyle factors (Andreu-Sánchez et al., 2022). Therefore, although shorter telomeres have been associated with AD and may reflect accelerated biological aging, telomere length should be interpreted cautiously as definitive marker of AD (Rodríguez-Fernández et al., 2025). Nevertheless, telomere erosion may contribute to neuronal vulnerability by impairing cellular resilience and amplifying oxidative and inflammatory pathways (Forero et al., 2016; Huang X. et al., 2025; Khurshid et al., 2025).

### 2.2 DNA methylation

Among the epigenetic mechanisms implicated in AD, DNA methylation represents a crucial process linking genetic susceptibility to environmental and aging-related factors (Figure 2; Qin et al., 2023; Tisato et al., 2023, 2024). This process has been widely investigated in the context of neurodegenerative disorders, including AD, where it plays a key role in disease pathogenesis (De Plano et al., 2024; Tiwari et al., 2025). DNA methylation, primarily involving the covalent addition of a methyl group to the 5-position of cytosine residues, generating 5-methylcytosine (5mC), is a stable and heritable modification catalyzed and regulated by DNA methyltransferases (DNMTs), which establish and modulate DNA methylation patterns (Tiwari et al., 2025; Kadar Shahib and Rastegar, 2026). 5mC can undergo oxidation by the ten-eleven translocation (TET) family of dioxygenases, generating 5-hydroxymethylcytosine (5hmC), 5-formylcytosine (5fC), and 5-carboxylcytosine (5caC). These modified cytosine derivatives represent distinct epigenetic marks involved in the regulation of gene expression and chromatin organization (Zhang X. et al., 2023). Specifically, 5hmC is highly abundant in the brain, especially in neurons, where it plays an important role in neuronal development and transcriptional regulation (Al-Mahdawi et al., 2014). Increasing evidence suggests that alterations in 5hmC may also contribute to AD pathogenesis (Zhao et al., 2025). Epigenome-wide association studies (EWAS) have demonstrated that patients with clinically diagnosed AD are characterized by an extensive remodeling of the DNA methylome compared with age- and sex-matched healthy controls, exhibiting overall global hypomethylation (Smith et al., 2021; Breen et al., 2023). However, analyses of differentially methylated regions (DMRs) have revealed consistent hypermethylation at specific genomic loci associated with AD pathology (Smith et al., 2021; Lukacsovich et al., 2025). In this regard, Lukacsovich et al. (2025) performed a meta-analysis of DNA methylation profiles in the prefrontal cortex using two independent post-mortem brain cohorts, the Religious Orders Study and Memory and Aging Project (ROSMAP) and Brains for Dementia Research (BDR). The authors identified 3,264 age-associated CpG sites, the majority of which were hypermethylated and enriched in promoter regions and CpG islands. Interestingly, these age-related methylation changes showed a remarkable overlap and concordant directionality with AD-associated DMRs, indicating that epigenetic alterations, accumulating during physiological aging, may contribute to the molecular mechanisms underlying AD progression, including immune regulation, synaptic function, and neuronal differentiation (Lukacsovich et al., 2025). This overlap between age-associated and AD-associated methylation changes is particularly relevant when considering late-onset AD (LOAD), in which advanced age represents the strongest risk factor, and supports the concept that at least part of the AD-associated methylome may reflect the interaction between pathological processes and the epigenetic consequences of normal aging (Pellegrini et al., 2021).

A further level of complexity lies in the regional heterogeneity of methylation profiles across the brain. Specifically, brain areas most vulnerable to AD (i.e., entorhinal cortex, hippocampus, prefrontal cortex, superior temporal gyrus) display the highest number of differentially methylated CpG sites/regions. In contrast, brain regions relatively spared by neurodegeneration show fewer alterations in DNA methylation. This spatial pattern closely parallels the distribution of Aβ plaques and neurofibrillary tangles, suggesting that region-specific epigenetic remodeling contributes to the selective vulnerability and progression of AD (Altuna et al., 2019; Alves et al., 2024). In addition, recent evidence has demonstrated that brain areas most affected by AD exhibit epigenetic aging acceleration through epigenetic clocks (Pellegrini et al., 2021; Thrush et al., 2022). Thrush et al. (2022) developed a principal component-based epigenetic clock (i.e., PCBrainAge) specifically for the human brain. The authors demonstrated that AD is associated with accelerated brain epigenetic aging specifically in AD-affected areas, supporting the concept that DNA methylation changes reflect the regional progression of neurodegeneration (Thrush et al., 2022). While EWAS have revealed extensive methylome remodeling in AD, they have also identified a limited number of consistently differentially methylated genes directly implicated in AD pathogenesis. The limited replication of differentially methylated genes across studies may reflect substantial biological and methodological heterogeneity, including differences in brain regions, disease stage, cohort characteristics, and analytical approaches (Nabais et al., 2021). Moreover, cellular heterogeneity and the cell-type- and region-specific nature of epigenetic alterations may further contribute to discrepancies across studies. Sex may also contribute to this heterogeneity, as some studies have reported sex-specific DNA methylation patterns in AD-associated loci (Zhang et al., 2021c). For example, sex-dependent methylation differences have been reported at genes involved in AD-related pathways, suggesting that epigenetic alterations may contribute differently to disease susceptibility and progression in males and females, although the available evidence remains limited (Zhang et al., 2021c). Importantly, the limited replication of individual differentially methylated genes across studies does not necessarily indicate a lack of biological relevance of DNA methylation in AD. Rather, it may reflect the complex and context-dependent nature of epigenetic alterations. Thus, differences between studies may partly reflect the specific anatomical and pathological context examined rather than completely inconsistent biological findings (Cheng et al., 2024). Future studies integrating multiple brain regions, longitudinal disease stages, cell-type-specific analyses, sex-stratified approaches, and standardized analytical pipelines may therefore be essential to distinguish robust disease-associated methylation changes from alterations related to aging, cellular composition, or disease progression (Smith et al., 2021). The principal site-specific DNA methylation alterations associated with AD-both hypermethylation and hypomethylation-are summarized in Table 1.

**TABLE 1:** DNA methylation changes in AD-associated genes and pathogenic mechanisms.

Gene	DNA methylation	Effect on gene expression	Relevance in AD	Sample	AD form	Species	References
*BIN1*	Hypomethylation	Altered *BIN1* expression	Decreased CSF Aβ42; increased p-tau/Aβ42 and t-tau/Aβ42	Blood	Preclinical LOAD	Human	Hu et al., 2021
*APOE*	Differential methylation at specific CpG sites; hypermethylation at two CpG sites	Increased *APOE* expression	Effects on Aβ clearance and lipid metabolism	Blood/brain	LOAD	Human	Panitch et al., 2024
*APP*	Hypomethylation	Increased *APP* transcription	Increased Aβ production	Brain	FAD model	Mouse	Park et al., 2022
*MAPT*	Hypomethylation	Increased *MAPT* expression	Increased tau pathology	Brain	Not specified	Human	Mori et al., 2022
*TREM2*	Hypomethylation	Increased *TREM2* expression	Associated with activated microglia	Blood	Not specified	Human	Hu et al., 2014
	Hypermethylation	Reduced *TREM2* expression	Impaired microglia phagocytosis and Aβ clearance	Brain (superior temporal gyrus)	Not specified	Human	Smith et al., 2016
*ABCA7*	Hypermethylation	Reduced *ABCA7* expression	Impaired lipid homeostasis and Aβ clearance	Brain	LOAD	Human	Yu L. et al., 2015
*BACE1*	Hypomethylation	Increased *BACE1* expression	Increased β-secretase and Aβ generation	Brain	LOAD	Human	Li P. et al., 2019
*BDNF*	Hypermethylation	Altered *BDNF* expression	Impaired synaptic plasticity, neuronal survival and cognitive function	Brain (prefrontal cortex, hippocampus)	Not specified	Human	Nagata et al., 2015
*PSEN1*	Hypomethylation	Increased *PSEN1* expression	Enhanced γ-secretase activity and Aβ production	Brain	LOAD/FAD model	Human/mouse	Monti et al., 2020
*NEP*	Hypermethylation	Reduce *NEP* expression	Reduced Aβ degradation and clearance	Brain	Not specified	Human	Nagata et al., 2018
*ANK1*	Hypermethylation	Altered *ANK1* expression	Increased neuroinflammation and microglial activation; impaired synaptic plasticity	Brain (entorhinal and prefrontal cortex, superior temporal gyrus)	LOAD	Human	Lunnon et al., 2014; Smith et al., 2019

### 2.3 RNA methylation

Although DNA methylation has been extensively investigated in AD, epigenetic regulation also occurs at the RNA level. This emerging layer of regulation, known as “epitranscriptomics,” has attracted increasing attention in neurodegenerative diseases because of its fundamental role in controlling neuronal gene expression and brain homeostasis (Paraboschi et al., 2014, 2015; Huang et al., 2020; Yao et al., 2025). Among RNA modifications, N6-methyladenosine (m6A) is the most abundant internal RNA methylation, representing approximately 80% of RNA methylation modifications in eukaryotes (Xia et al., 2023; Hu et al., 2025). m6A plays a significant role in brain development and function as a post-transcriptional regulator involved in neural development, synaptic transmission, and postsynaptic function (Huang et al., 2020). Evidence indicates that the dysregulation of the m6A machinery is involved in AD pathogenesis. Overall, a decrease in m6A methylation levels is observed in the brain of AD patients compared to cognitively intact older adults, particularly evident in large pyramidal neurons crucial for memory, learning, and information integration (Castro-Hernández et al., 2023). In addition, the hippocampus of AD patients exhibited a decreased level of the m6A methylases, including methyltransferase-like protein 3 (METTL3) and METTL14. In support of this, METTL3 and METTL14 deficient mice show impaired synaptic plasticity, memory, and learning. Conversely, METTL3 expression is up-regulated in microglia, which activates the TRAF6/NF-κB signaling pathway and contributes to neuroinflammation (Wen et al., 2022). In addition, patients with AD exhibit a loss of the proline rich coiled-coil 2 A (PRRC2A), a m6A reader highly expressed in oligodendrocytes, resulting in oligodendrocyte dysfunction and demyelination, early events observed in AD white matter damage (Wu et al., 2019). In a recent study, Hu et al. (2025) profiled the RNA m6A methylome in the frontal cortex of post-mortem brains from individuals with or without AD. The study demonstrated that m6A positively regulates the expression of promoter antisense RNAs (paRNAs), including a MAPT-associated paRNA (MAPT-paRNA) that is highly expressed in neurons. This ncRNA promotes the expression of broad neuronal and synaptic genes (Hu et al., 2025). Taken together, these data support the emerging role of m6A in several cellular processes underlying AD pathogenesis and progression.

### 2.4 Histone modifications

Histone post-translational modifications (hPTMs) are key epigenetic regulators that dynamically regulate chromatin organization and accessibility, and thereby affect transcriptional activity by altering histone–DNA interactions and recruiting regulatory proteins to specific genomic regions (Pal and Tyler, 2016). hPTMs have been mainly associated with learning, memory, synaptic plasticity, and cognitive functions, and the dysregulation of these processes was found in mouse models of aging and AD (Figure 2; Santana et al., 2023). Human large-scale epigenome studies focusing on hPTMs in AD have demonstrated that, while there is a loss of some histone marks, there is also a gain of other marks, supporting the complex dynamics of histone modifications in AD (Nativio et al., 2020). The most common modifications include methylation, acetylation, and ubiquitination. Figure 3 schematically illustrates the major hPTMs commonly observed in patients with AD, classified according to their role in disease pathogenesis.

**FIGURE 3:**
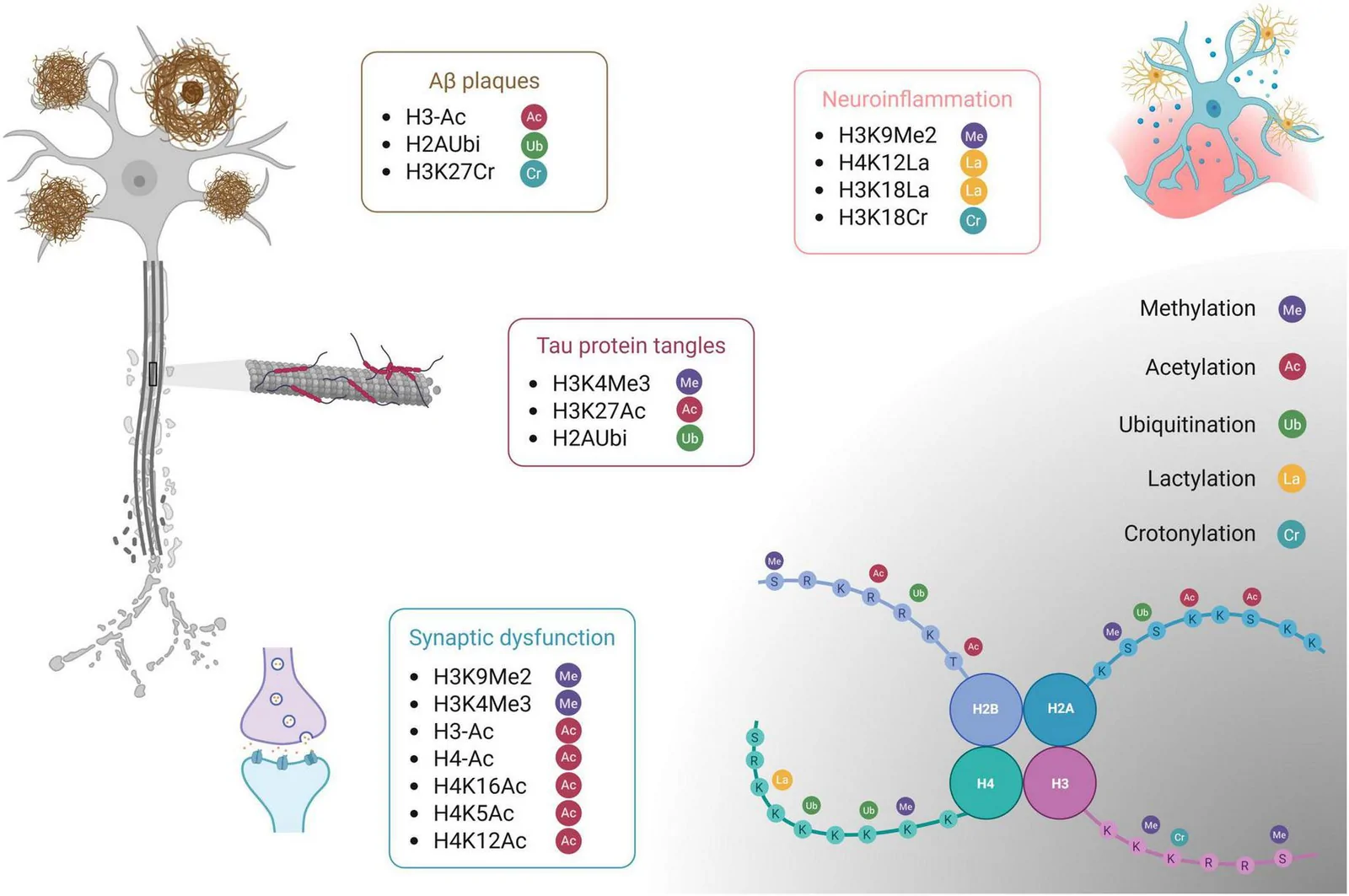
Overview of histone modifications associated with AD pathological processes. Altered histone methylation (Me), acetylation (Ac), ubiquitination (Ub), lactylation (La), and crotonylation (Cr) contribute to transcriptional dysregulation underlying amyloid-β (Aβ) plaque formation, tau protein aggregation, neuroinflammation, and synaptic dysfunction.

One of the alterations found abnormally elevated in post-mortem prefrontal cortex and hippocampus of AD patients is the dimethylation of H3 lysine 9 (H3K9me2), which acts as a repressive epigenetic mark that silences essential genes involved in synaptic plasticity and neurotransmission, exacerbating neuroinflammatory responses by influencing microglial activation via GLP/G9A methyltransferase complex (Bellver-Sanchis et al., 2024, 2026). The histone mark H3K4me3 and its catalytic enzymes- SETD1a/b and MLL1–4- are elevated in the prefrontal cortex and hippocampus of patients with AD. In particular, increased H3K4me3 enrichment at specific gene loci, such as SGK1, results in increased levels of hyperphosphorylated tau and altered synaptic functions (Cao et al., 2020; Li et al., 2025). Numerous studies have demonstrated that AD is also associated with profound alterations in histone acetylation patterns, particularly at H3 and H4, which primarily regulate genes involved in synaptic plasticity, as well as accelerate Aβ secretion and tau protein tangle formation (Narayan et al., 2015; Klein et al., 2019; Nativio et al., 2020; Basavarajappa and Subbanna, 2024). The overall increase in H3 and H4 acetylation appears to be region-specific, occurring mainly in brain areas exhibiting severe pathological AD hallmarks, while relatively unaffected regions show no significant changes (Qin et al., 2024). Acetylation imbalance impairs neuronal transcriptional programs leading to synaptic disruption, mainly through dysregulation of glutamatergic signaling, and indirectly contributing to increased expression of amyloidogenic enzymes (e.g., BACE1, γ-secretase) (O’Connor et al., 2020). Specifically, H3K27Ac levels were significantly enriched in regulatory regions proximal to the *MAPT* gene, which encodes tau protein and is associated with active transcription (Marzi et al., 2018). Of particular interest is the role of acetylation at specific lysine residues of histone H4. The chromatin state, through the mark H4K16Ac, has been implicated in healthy aging. In age-associated neurodegenerations, including AD, the mark is dramatically lost, driving synaptic dysfunctions and cognitive decline (Nativio et al., 2018). Similarly, H4K5Ac and H4K12Ac are significantly reduced at the promoter regions of essential synaptic plasticity genes (e.g., *BDNF*, *GRIN1*) (Li et al., 2025; Chai et al., 2026). The monoubiquitination of histone H2A (H2AUbi) acts as an epigenetic brake that turns off specific genes, preventing them from being expressed (Bae et al., 2025; Mishra et al., 2026). In the context of AD, the levels of this repressive histone mark are reduced in AD brain, contributing to accumulation of neurotoxic proteins such as Aβ and hyperphosphorylated tau (Guo et al., 2022). Finally, histone lactylation and crotonylation are two recently identified hPTMs each involving the enzymatic transfer of a lactyl or crotonyl group, respectively, to lysine residues (Xie et al., 2022; Weng et al., 2026). *Wei et al. (2023)* mapped all histone lactylation sites in senescent brain and found that H3K18La was significantly up-regulated in the hippocampus of mouse models of AD. They also demonstrated that enhanced H3K18La directly stimulates the NFκB signaling pathway by increasing binding to the promoter of Rela (p65) and NFκB1 (p50), leading to neuroinflammation (Wei et al., 2023). Likewise, H4K12La was found to be significantly elevated in microglia of AD mice and patients, and contributes to pro-inflammatory activation (Pan et al., 2022). H3K18Cr has also been implicated in AD-related neuroinflammation, although through a different mechanism. Experiments in AD mouse models have shown that elevated mark levels found in microglia promote the activation of the transcription factor STAT1, thereby inducing the expression of downstream immune-related genes (Weng et al., 2026). In addition to promoting neuroinflammation, histone crotonylation has also been implicated in Aβ plaque clearance. Specifically, in AD, elevated levels of H3K27Cr have been detected next to transcription start sites of different genes, including those involved in endocytosis. H3K27Cr was found to be influenced by the long non-coding NEAT1, which interacts with the p300/CBP complex, ultimately leading to endocytosis blockage (Wang Z. et al., 2019). Sex may also influence histone modification patterns in AD. For example, a genome-wide analysis of H3K4me3 and H3K27me3 in the entorhinal cortex of AD patients showed a reduction in H3K4me3 and an increase in H3K27me3 in AD; notably, the increase in H3K27me3 was observed in male but not female patients, suggesting sex-dependent epigenetic responses to AD pathology (Persico et al., 2022). These findings highlight the potential contribution of sex to histone remodeling in AD, although further studies are needed to clarify the underlying mechanisms and their functional relevance.

### 2.5 Mitochondrial DNA methylation

The burgeoning field of mitoepigenetics (mitochondrial epigenetics) has emerged as a potential contributor to age-related diseases, including AD (Figure 2; Neikirk et al., 2025). Mitochondria are considered the primary energy hubs of eukaryotic cells and are key regulators of redox homeostasis, calcium signaling and apoptosis; therefore, mitochondrial aging may contribute to metabolic imbalance and exacerbate oxidative stress and related pathways, which drive neurodegenerative processes (Meng et al., 2025). Unlike nuclear DNA, mtDNA lacks canonical histones and introns. Consequently, the regulation of mitochondrial gene transcription relies on epigenetic modifications. Among these mechanisms, mtDNA methylation represents the most extensively investigated epigenetic modification. However, the presence and functional relevance of mtDNA methylation remain controversial. Reported methylation levels vary considerably depending on the tissue, genomic region, and methodological approach. In particular, the relative contribution of CpG versus non-CpG methylation remains incompletely defined, as does the strand specificity of mtDNA methylation (Stoccoro and Coppedè, 2021). Furthermore, the distinction between 5mC and 5hmC, as well as the mitochondrial localization and functional activity of the DNMTs and TETs remain elusive (Shock et al., 2025). Methodological limitations, particularly those associated with bisulfite-based approaches, may also contribute to discrepancies among studies, as these methods can be affected by incomplete conversion and cannot reliably distinguish 5mC from 5hmC (Nestor et al., 2010). Nevertheless, methylation in mtDNA, particularly of the displacement loop (D-loop), has been proposed to contribute to structural and functional stability of the genome and may be involved in the regulation of mitochondrial gene transcription, replication, and mtDNA copy number (Cavalcante et al., 2020; Stoccoro et al., 2021). Aberrant mtDNA methylation disrupts mitochondrial homeostasis, leading to impairment of key mitochondrial functions. An investigation of the entorhinal cortex and substantia nigra of post-mortem brains of early-stage AD cases has demonstrated that DNA methylation of CpG and non-CpG sites is increased in the D-loop region, while it is decreased in NADH dehydrogenase subunit 1 (ND1) (Blanch et al., 2016; Stoccoro et al., 2022). Changes in mtDNA methylation have also been detected in peripheral blood, suggesting their potential utility as minimally invasive biomarkers for early diagnosis and disease progression. Accordingly, *Ding et al. (2023)* found that the global methylation level of cell-free mtDNA was lower in patients with AD than in matched healthy subjects, and that mtDNA hypomethylation was mainly located in non-protein coding regions of mitochondrial genome. Conversely, in the mitochondrial protein-coding region, hypermethylation of mt-ND4 was observed only in AD patients (Ding et al., 2023). These findings indicate that mtDNA methylation is dynamically regulated across different tissues and disease stages, although the biological significance of these changes need to be further studies to link aging, energy metabolism, and neurodegeneration in AD. Further studies using orthogonal, methylation-sensitive approaches capable of distinguishing 5mC from 5hmC and resolving strand-specific methylation, together with functional experiments, are needed to establish the clear biological significance of mtDNA methylation and its potential role in linking aging, energy metabolism, and neurodegeneration in AD.

### 2.6 Non-coding RNAs

ncRNAs are considered one of the most promising classes of epigenetic biomarkers in AD, finely regulating gene expression at transcriptional and post-transcriptional levels (Figure 2; Salvatori et al., 2023; D’Aversa et al., 2024). The three major classes of ncRNAs are micro RNAs (miRNAs), long non-coding RNAs (lncRNAs), and circular RNAs (circRNAs), which differ both structurally and functionally (Diamantopoulos et al., 2025; Ahim et al., 2026). Accumulating evidence demonstrates that several ncRNAs are aberrantly expressed in the different areas of the brain and peripheral fluids of AD patients, where they are closely associated with key pathological processes, including Aβ production and clearance, tau hyperphosphorylation, synaptic dysfunction, and neuroinflammation, contributing to the complex molecular network underlying neurodegeneration (Zhang et al., 2021d; Alzarea, 2025). Their expression profiles also appear to correlate with disease severity and progression, suggesting potential diagnostic and prognostic value (Canoy et al., 2024; Sun et al., 2026).

#### 2.6.1 Micro RNAs

Among ncRNAs, miRNAs are the most extensively characterized. miRNAs are a class of small RNA molecules, ranging in length from 20 to 24 nucleotides, that negatively regulate gene expression (Salvatori et al., 2023; Li Y. B. et al., 2024). miRNAs act through two primary mechanisms: (i) by binding completely to the 3′ untranslated region (3′ UTR) of target mRNAs, leading to mRNA degradation and gene expression inhibition; (ii) by partially binding to the 3′ UTR to repress the translation process, thereby reducing gene expression (O’Brien et al., 2018; Li Y. B. et al., 2024). miRNAs dysregulation has been implicated in the pathogenesis of various diseases, including neurodegenerative disorders like AD (Kashif and Said, 2025). Specifically, in AD, several miRNAs have been identified as key epigenetic regulators of disease pathological mechanisms, including Aβ deposition, tau phosphorylation, inflammation, oxidative stress, genetics, and mitochondrial dysfunction (Miya Shaik et al., 2018; Li Y. B. et al., 2024; Sharma et al., 2024). An overview is presented in Figure 4.

**FIGURE 4:**
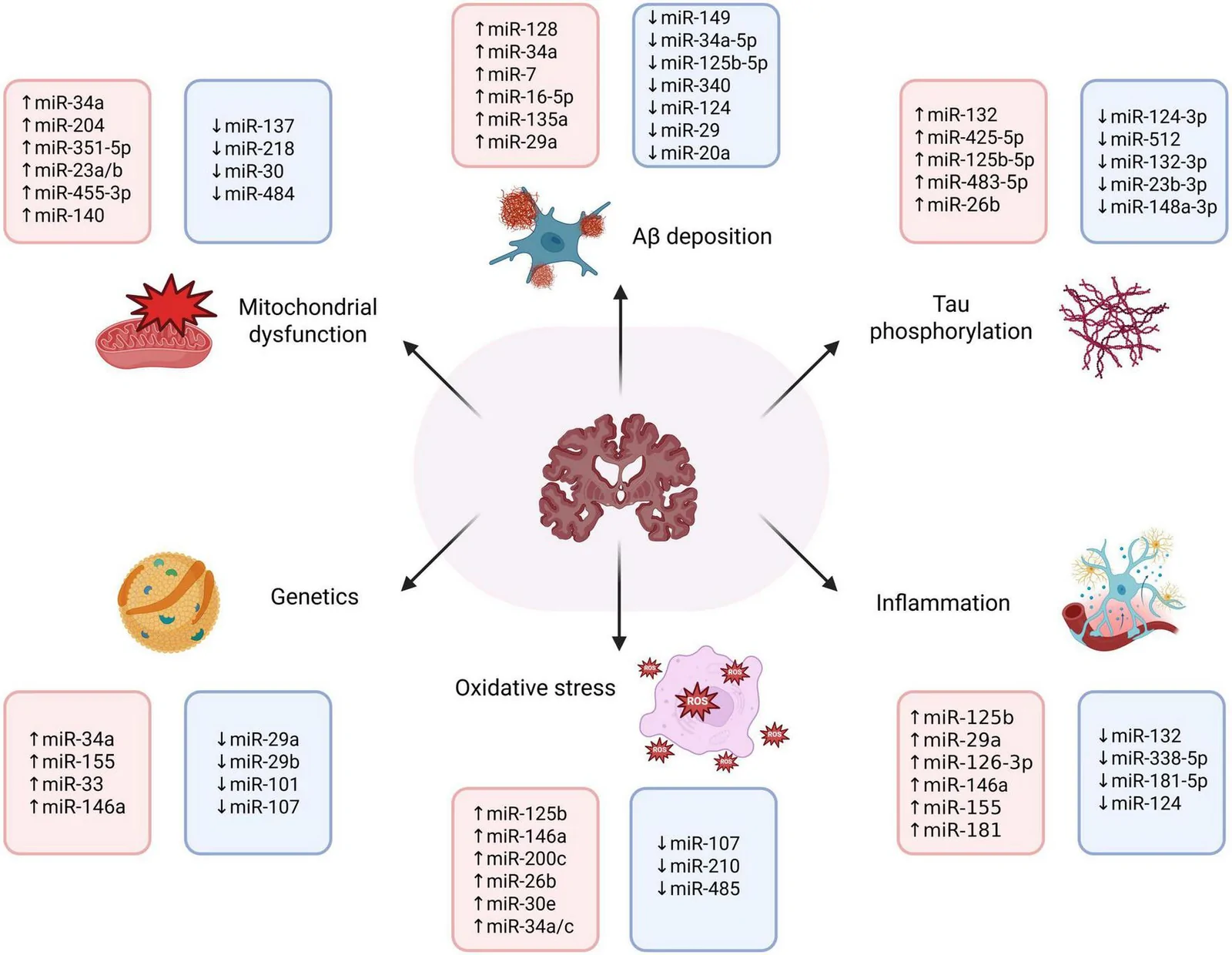
Overview of dysregulated micro RNAs involved in AD pathogenic mechanisms. The figure presents the main miRNAs (miR) reported to be upregulated (↑) or downregulated (↓) associated with the key pathological processes of AD, including amyloid-β (Aβ) deposition, tau phosphorylation, inflammation, oxidative stress, genetics, and mitochondrial dysfunction.

##### 2.6.1.1 miRNAs in Aβ deposition

Aβ deposition results from an imbalance between Aβ production and clearance (Ahmadi et al., 2025). Several miRNAs regulate this process by targeting genes involved in APP processing, particularly *BACE1* and *APP*, and signaling pathways that influence Aβ metabolism (Li Y. B. et al., 2024). Among the dysregulated miRNAs in AD, miR-128 has been shown to suppress Aβ accumulation by directly inhibiting the expression of APPBP2 and mTOR, thereby reducing Aβ generation (Li et al., 2023). miR-34a is associated with increased BACE1 expression and Aβ-induced neurotoxicity (Sarkar et al., 2019). miR-7, upregulated in AD cortical tissues, contributes to neuronal dysfunction by modulating pathways involved in APP metabolism (Fernández-de Frutos et al., 2019), whereas miR-16-5p and miR-135a regulate APP-related signaling and neuronal viability (Kim et al., 2020; Zheng et al., 2021). miR-29a is frequently dysregulated in AD cerebrospinal fluid (CSF) during AD progression and participates in the regulation of BACE1 expression (Hébert et al., 2008). Interestingly, miRNA-29a has also been implicated in the aging process and aging-related phenotypes (Swahari et al., 2024). Conversely, downregulated miRNAs, including miR-149, miR-34a-5p, miR-125b-5p, miR-340, miR-124, miR-20a, and miRNAs belonging to the miR-29 family, normally exert protective effects by suppressing BACE1 or APP expression and promoting Aβ clearance (An et al., 2017; Zhao et al., 2017; Li P. et al., 2020; Du et al., 2021; Li Y. B. et al., 2024). Their reduced expression, predominantly in frontal cortex, removes this inhibitory control, leading to enhanced Aβ production and plaque formation (Lei et al., 2015; Li P. et al., 2020; Abuelezz et al., 2021; Du et al., 2021).

##### 2.6.1.2 miRNAs in tau phosphorylation

Abnormal tau phosphorylation leads to neurofibrillary tangle formation and progressive neuronal dysfunction (Ahmadi et al., 2025). Several miRNAs regulate tau pathology by modulating MAPT expression or the activity of tau-related kinases, including GSK-3β, CDK5, and ERK (Wang et al., 2023). miR-132, miR-425-5p, miR-125b-5p, miR-483-5p, and miR-26b levels were found to be upregulated in AD patients and have been associated with enhanced tau phosphorylation through the activation of pro-phosphorylation pathways and the dysregulation of neuronal signaling (Absalon et al., 2013; Banzhaf-Strathmann et al., 2014; El Fatimy et al., 2018; Yuan et al., 2020; Nagaraj et al., 2021). Specifically, miR-125b promotes tau hyperphosphorylation by suppressing the phosphatases DUSP6 and PPP1CA, thereby increasing CDK5 and MAPK activity (Banzhaf-Strathmann et al., 2014). miR-26b induces aberrant cell-cycle re-entry and activates tau kinases (Absalon et al., 2013). Similarly, miR-132 and miR-425-5p overexpression alter tau metabolism, activating GSK-3β (Yuan et al., 2020; Li Y. B. et al., 2024). On the other hand, in a physiological context, miR-124-3p, miR-512, miR-132-3p, miR-23b-3p, and miR-148a-3p offer a neuroprotective effect by inhibiting abnormal tau hyperphosphorylation. In the context of AD, these specific miRNAs are downregulated, and their loss removes inhibitory control over tau-related signaling pathways, thereby favoring tau hyperphosphorylation and the formation of neurofibrillary tangles (Nagaraj et al., 2021; Zhao H. et al., 2021).

##### 2.6.1.3 miRNAs in inflammation

Inflammation in AD is primarily driven by microglial and astrocyte activation, leading to the release of pro-inflammatory cytokines that exacerbate neuronal dysfunction and neurodegeneration (Sharma et al., 2026). Several miRNAs (inflamma-miRNAs) modulate inflammatory signaling pathways, including NF-κB, TLR4, and the NLRP3 inflammasome (Liang and Wang, 2021). Inflamma-miRNAs are found to be dysregulated in AD contributing to neuroinflammatory processes (Slota and Booth, 2019; Liang and Wang, 2021). Among the most consistently upregulated miRNAs, miR-125b, miR-29a, miR-126-3p, miR-146a, miR-155, and miR-181 are associated with sustained neuroinflammation. More in depth, miR-155 is a key pro-inflammatory regulator that enhances microglial activation and cytokine production (Paraboschi et al., 2011; Zingale et al., 2021), while miR-146a, although initially induced as a negative feedback modulation of the NF-κB pathway, becomes chronically dysregulated in AD and contributes to persistent inflammatory responses (Long et al., 2024). miR-125b promotes inflammatory signaling and neuronal injury by targeting anti-inflammatory and synaptic genes (e.g., *SYN-2*) (Lukiw, 2012; Liu W. et al., 2024), whereas miR-29a and miR-181 have been implicated in immune dysregulation during AD progression, regulating inflammatory and cellular stress responses through targets such as Fos and SIRT1 (Rodriguez-Ortiz et al., 2014; Mei et al., 2024). Conversely, miR-132, miR-338-5p, miR-181-5p, and miR-124 are downregulated in AD. Specifically, miR-132 is one of the most significantly downregulated miRNAs in AD. Supplementation with miR-132 has been shown to reduce pro-inflammatory signatures in microglia and promote neuronal survival (Penning et al., 2026). miR-338-5p is significantly reduced in the AD hippocampus. In a physiological context, it suppresses the enzyme BACE1 and reduces neuroinflammation, protecting synaptic plasticity and memory (Qian et al., 2019). miR-181c-5p downregulation in AD patients impairs the ability of microglial cells to clear toxic Aβ plaques. Accordingly, its restoration successfully alleviates neuroinflammation (Li R. et al., 2024). miR-124 is involved in the pathogenesis of AD through various mechanisms including being a critical regulatory factor in the hypoxia/Aβ-miR-124-BACE1-Aβ cycle. In addition, miR-124 expression was downregulated with aging (Zhao J. et al., 2021).

##### 2.6.1.4 miRNAs in oxidative stress

Oxidative stress is a central feature of AD pathogenesis and results from excessive production of ROS, leading to lipid peroxidation, protein oxidation, mitochondrial damage, and ultimately neuronal loss (Alkhalifa et al., 2025). Oxidative stress modulates the expression of various miRNAs, which in turn regulate genes integral to the oxidative stress response, including cellular antioxidant defense system (Li Y. B. et al., 2024). Upregulated miRNAs associated with AD-related oxidative stress include miR-125b, miR-146a, miR-200c, miR-26b, miR-30e, and miR-34a/c. miR-125b promotes ROS production and neuronal apoptosis by modulating pro-survival signaling pathways (Jin et al., 2018), while miR-146a is induced by oxidative stress and chronic NF-κB activation, contributing to persistent inflammation and redox imbalance by downregulating SOD2 (Zhao et al., 2023). miR-200c is activated in hippocampal neurons in response to oxidative injury and regulates apoptosis-related genes (e.g., *Bax*, *Bim*) (Yu D. S. et al., 2015; Wu et al., 2016), whereas miR-26b is upregulated in the temporal cortex and promotes oxidative stress via targeting RB1, which drives aberrant cell cycle entry and activation of CDK5 (Absalon et al., 2013). miR-30e is upregulated in the hippocampus and increases the levels of SOD, GSH, and GSH-PX and decreases ROS levels by targeting SNAI1 through decreasing TGF-β and SMAD2 expression (Nunomura and Perry, 2020). miR-34a and miR-34c are upregulated in the AD brain and reduce tolerance to oxidative stress by targeting SIRT1, thereby increasing neuronal vulnerability (Choi and Kemper, 2013; Sarkar et al., 2019). In contrast, miR-107, miR-210, and miR-485 are downregulated in AD. These miRNAs normally protect neurons by regulating mitochondrial metabolism, antioxidant defenses, and cell survival; therefore, their reduced expression leads to NMDAR overactivation, excessive calcium influx, mitochondrion-derived ROS production, mitochondrial dysfunction, and oxidative neuronal damage, ultimately accelerating aging (Nunomura and Perry, 2020; Koh et al., 2021; Subasinghe et al., 2025).

##### 2.6.1.5 miRNAs in genetics

As previously reported, genetic susceptibility plays a crucial role in AD onset and progression (Tisato et al., 2018; Qi et al., 2025). Several miRNAs contribute to disease pathogenesis by modulating the expression of AD-associated genes, including *APP, BACE1, PSEN1/2*, and *APOE* (Yesudas et al., 2025). miR-29a, miR-29b, miR-101 and miR-107 negatively regulate BACE1. Consequently, reduced expression of these miRNAs in AD enhances the amyloidogenic processing of APP (Wang M. et al., 2019; Li Y. B. et al., 2024). Otherwise, elevated levels of miR-34a, miR-155, miR-33, and miR-146a have been found in AD. In detail, miR-34a regulates *APP*, *BACE1*, and *APOE* expression (Sarkar et al., 2019). miR-33 regulates cholesterol homeostasis and lipid metabolism, processes associated with APOE functions (Kim et al., 2015), while miR-146a modulates APP and TSPAN12, influencing AD progression by activating the amyloidogenic pathway (Long et al., 2024).

##### 2.6.1.6 miRNAs in mitochondrial dysfunctions

Alteration in mitochondrial homeostasis is an early event in AD and contributes to impaired ATP production, calcium dyshomeostasis, excessive ROS production, and neuronal apoptosis (Zhu et al., 2013; D’Alessandro et al., 2025). Notably, age-related mitochondrial dysfunction represents a fundamental hallmark of brain aging and is considered one of the major contributors to AD pathogenesis (Bartman et al., 2024). During aging, mitochondria progressively accumulate mtDNA damage, reduce respiratory efficiency, impair mitophagy, and increase oxidative stress, thereby creating a cellular environment that predisposes to neurodegeneration (Subasinghe et al., 2025). Several miRNAs regulate mitochondrial homeostasis by targeting genes involved in mitochondrial quality control, including mitophagy, biogenesis, dynamics, oxidative phosphorylation, and apoptosis (Li et al., 2012; Zhang et al., 2021a). Among the upregulated miRNAs in AD, miR-34a is one of the well-characterized regulators, suppressing SIRT1 and BCL2, thereby increasing oxidative stress and neuronal apoptosis (Sarkar et al., 2016). Upregulation of miR-204 in hippocampal neurons has been implicated in mitochondrial metabolic dysregulation and oxidative injury via the STAT3/TRPML1 pathway (Zhang et al., 2021b). Emerging evidence suggests that miR-455-3p, miR-140, miR-23a/b, and miR-351-5p may further influence mitochondrial dynamics, bioenergetics, and apoptotic pathways, although their precise roles in AD remain under investigation (Rivera et al., 2023; Islam et al., 2024; Subasinghe et al., 2025). Conversely, miR-484, miR-137, miR-30, and miR-218 act as neuroprotective epigenetic molecules, as they contribute to the maintenance of mitochondrial integrity/homeostasis and quality control (Channakkar et al., 2020; Rosolen et al., 2023). Specifically, alterations in mitochondrial dynamics (i.e., fusion and fission) are associated with the downregulation of miR-30 and miR-484 (Wang et al., 2012; Gowda et al., 2022).

#### 2.6.2 Circular RNAs

Circular RNAs have emerged as a novel class of endogenous RNAs with important regulatory functions in the central nervous system (Li et al., 2021; Cardamone et al., 2023; Baiddou et al., 2026). Unlike linear RNAs, circRNAs are generated through back-splicing events, forming covalently closed-loop structures that confer remarkable stability and resistance to exonuclease-mediated degradation (D’Aversa et al., 2024). These distinctive characteristics, together with high tissue specificity, make them unique and minimally invasive biomarkers useful for early disease diagnosis and prognosis (Baiddou et al., 2026). circRNAs are highly abundant in the brain, where their exceptional stability favors their accumulation in long-lived neurons. Furthermore, they display cell type- and developmental stage-specific expression patterns, and are particularly enriched at synapses, where they contribute to neuronal differentiation, synaptic plasticity, and cognitive function (Huang S. et al., 2025). At the molecular level, circRNAs regulate gene expression through multiple mechanisms, including (i) acting as miRNA sponges, (ii) interacting with RNA-binding proteins, (iii) modulating transcription, and (iv) serving as templates for peptide translation (D’Aversa et al., 2024). Through these diverse regulatory functions, circRNAs play a key role in maintaining neuronal homeostasis, and their dysregulation may therefore contribute to the molecular alterations underlying neurodegeneration (Ziaei et al., 2026). Dysregulation of circRNAs is involved in the pathogenesis of AD through the modulation of multiple molecular pathways, including Aβ metabolism, tau pathology, neuroinflammation, oxidative stress, and neuronal survival (Beylerli et al., 2023; Puri et al., 2023; Phillips et al., 2026). Table 2 summarizes the main human circRNAs associated with AD pathogenesis, highlighting the altered expression levels, the known molecular targets, the pathogenic mechanisms and the biological samples in which they are identified.

**TABLE 2:** Circular RNAs involved in AD pathogenesis.

hsa_circRNA	Regulation	Target	Mechanism in AD	Sample	AD form	Species	References
circHDAC9	Down	miR-138	Reverses the Sirt 1 inhibition and increases Aβ overproduction	Serum	LOAD/FAD model	Human/mouse	Lu et al., 2019
circ0007556	Up	–	Translates into Aβ175 polypeptide, increasing Aβ biogenesis	Brain	Not specified	Human	Mo et al., 2020
circCwc27	Up	Pur-α	Increases level of Aβ40 and Aβ42	Brain	LOAD/FAD model	Human/mouse	Song et al., 2022
circ002048	Down	miR-422a, miR-4784, miR-3944-3p	Inhibits the expression of AP2M1 that contributes to tau hyperphosphorylation	Blood	Not specified	Human	Zhang et al., 2022
circPCCA	Down	miR-138-5p	Promotes phosphorylation of tau	CSF	Not specified	Human	Li P. et al., 2020
circ0005835	Up	miR-576-3p	Promotes Aβ-induced neuroinflammatory cytokines release	Serum	Not specified	Human/mouse	Xu et al., 2022
circHUWE1	Up	miR-433-3p	Promotes inflammatory progression and inhibits the activity of the Wnt/β-catenin signaling pathway	Serum	Not specified	Human	Abuduwaili et al., 2025
circ0049472	Up	miR-107	Enhances KIF1B expression by sequestering miR-107, thereby inhibiting cell viability and facilitating apoptosis, inflammation and oxidative stress	CSF, serum	Not specified	Human	Zeng et al., 2022
circ-0003611	Up	miR-383-5p	Increases apoptosis and neuronal injury triggered by Aβ	Serum	Not specified	Human	Li et al., 2022
circLPAR1	Up	miR-212-3p	Increases Aβ-induced neuronal apoptosis, oxidative stress and inflammation	Serum	Not specified	Human	Wu et al., 2021

#### 2.6.3 Long non-coding RNAs

LncRNAs are long RNA transcripts (>200 nucleotides in length) that lack protein-coding potential but exert important epigenetic regulatory functions at transcriptional and post-transcriptional levels (Zhang et al., 2019; Wu et al., 2024). Increasing evidence indicates that several lncRNAs contribute to the pathogenesis of AD by modulating key disease-associated pathways, including accumulation of Aβ/p-tau and pro-inflammatory activity (Wu et al., 2024). Among the well-characterized lncRNAs, BACE1-AS, found to be upregulated in different post-mortem brain tissues from AD patients, stabilizes BACE1 mRNA, thereby increasing BACE1 expression and promoting amyloidogenic APP processing and Aβ production (Li F. et al., 2019; Zeng et al., 2019). NEAT1 is another noteworthy disease-associated lncRNA, whose expression is elevated in the AD temporal cortex and hippocampus (Spreafico et al., 2018). NEAT1 regulates different miRNA-protein axes involved in the accumulation of Aβ/p-tau (e.g., NEAT1/miR-27a-3p/BACE1; NEAT1/miR-107), oxidative stress and mitochondria damage (e.g., miR-193a/CREB/BDNFaxis), and neuroinflammation (e.g., NEAT1/miR-291a-3p/Rab22a/NF-κB; miR-124/STAT3) (Dong et al., 2021; Liu Q. et al., 2022; Liu Q. et al., 2022; Li and Wang, 2023; Wu et al., 2024; Zamanian et al., 2026). MEG3 is upregulated in human neurons in AD and promotes activation of the RIPK1-RIPK3-MLKL necroptotic pathway, thereby contributing to neuronal cell death (Balusu et al., 2023). GAS5 acts as a molecular sponge for miRNA-23b-3p, which leads to the upregulation of downstream targets like GSK-3β and PTEN. This signaling cascade drives p-tau, Aβ buildup, and synaptic dysfunction (Zeng et al., 2026). Conversely, MALAT1 is emerging as a potential neuroprotective regulator in AD. MALAT1 is downregulated in CSF and blood of AD patients and has been shown to inhibit neuronal apoptosis and neuroinflammation. Mechanistically, the MALAT1/miR-125b axis exerts these effects by regulating downstream targets including PTGS2, CDK5, and FOXQ1 (Ma et al., 2019; Zhuang et al., 2020). These findings suggest that lncRNA dysregulation represents an important epigenetic mechanism contributing to the pathogenesis and progression of AD.

## 3 Epigenetic modifications as diagnostic, prognostic and therapeutic biomarkers of AD

In the first part of this review, we highlighted the emerging role of epigenetic alterations in the pathophysiology of AD and their association with key mechanisms underlying disease progression. This section explores the translational potential of epigenetic alterations, with a particular focus on their application as diagnostic and prognostic biomarkers and their emerging role as therapeutic targets in AD.

### 3.1 Diagnostic and prognostic relevance

Epigenetic dysregulation is emerging as a potentially informative molecular layer for refining the diagnosis, prognosis, and biological stratification of AD. Beyond their established contribution to AD pathogenesis- as discussed in the previous section- disease-associated alterations in DNA methylation and ncRNA profiles may capture dynamic molecular changes occurring across the continuum from preclinical disease to clinically manifest dementia (Fransquet and Ryan, 2019; Behl et al., 2024). Notably, several studies have identified epigenetic signatures in peripheral blood that are associated with AD diagnosis, established pathological biomarkers, or subsequent cognitive decline, raising the possibility of developing minimally invasive biomarkers for disease detection and monitoring (Roubroeks et al., 2020; Zhang W. et al., 2023). Alterations affecting AD-relevant genes and regulatory regions, including *PM20D1, HOXA5, HOXB6, ABCA7*, and other loci involved in amyloid processing, tau pathology, neuroinflammation, and synaptic function, further support the biological plausibility of these signatures (Roubroeks et al., 2020; Wang et al., 2020; Acha et al., 2023; Zhang W. et al., 2023). Importantly, longitudinal evidence suggests that selected epigenetic changes may precede clinical conversion, highlighting their potential utility for identifying individuals at increased risk before overt cognitive impairment becomes apparent (Wang et al., 2020; Pérez et al., 2022). Multimarker approaches integrating CpG methylation signatures or circulating ncRNAs may further enhance predictive performance compared with single molecular features (Fransquet and Ryan, 2019; Behl et al., 2024). Moreover, future biomarker strategies will likely rely on the integration of epigenetic profiles with established amyloid- and tau-based biomarkers, genetic risk factors, and clinical measures, rather than on isolated epigenetic signatures (Zhang W. et al., 2023). Nevertheless, translation into clinically actionable biomarkers remains challenging, owing to substantial heterogeneity in cohorts, biological tissues, analytical platforms, disease definitions, and statistical methodologies. Peripheral epigenetic profiles are also influenced by cellular composition, aging, genetic background, sex, and environmental or lifestyle exposures, which may limit their disease specificity (Di Francesco et al., 2015; Fransquet and Ryan, 2019). A critical unresolved issue is whether disease-associated epigenetic signatures represent causal mechanisms, downstream consequences of neurodegeneration, or molecular correlates of aging and systemic disease, a distinction that will be crucial for determining their diagnostic specificity and therapeutic relevance (Sanchez-Mut et al., 2018; Wang et al., 2020). Addressing this question, together with robust external validation, harmonization of analytical workflows, and prospective longitudinal studies in biomarker-defined populations, will be essential to establish the clinical utility of epigenetic biomarkers (Fransquet and Ryan, 2019). Some of the most relevant epigenetic biomarkers investigated for AD diagnosis and prognosis are summarized in Table 3.

**TABLE 3:** Diagnostic and prognostic biomarkers in AD.

Biomarkers	Diagnostic/ prognostic	Study cohort	Sample size	Sample	AD form	Species	Assay/platform	References
*NXN/ABCA7/HOXA3* methylation	Diagnostic	iBEAS case-control cohort	80 AD, 100 controls	Blood leukocyte	LOAD	Human	Bisulfite pyrosequencing	Acha et al., 2023
*ANKH/MARS* methylation	Diagnostic	Japanese discovery/validation study	Discovery: 12 AD, 12 controls; validation: 192 independent sample	Blood	Not specified	Human	Discovery: methylation capture sequencing; validation: bisulfite amplicon sequencing	Mitsumori et al., 2025
*HOXB6* hypermethylated DMR	Diagnostic	Multicenter European cohort	86 AD, 89 controls, 109 MCI	Blood	Not specified	Human	EWAS array with pyrosequencing validation	Roubroeks et al., 2020
*HOXA5* methylation	Diagnostic	ADNI with London neuropathology validation	202 in ADNI; 69 in London dataset	Pre-mortem blood vs. post-mortem brain in validation set	Not specified	Human	Illumina EPIC array	Zhang W. et al., 2023
miRNA panel	Diagnostic	Multiple blood/CSF studies summarized across independent cohorts	137 blood-miRNAs and 166 CSF-miRNAs datasets	Blood, CSF, brain	Not specified	Human	qPCR/sequencing platforms	Nagaraj et al., 2019
*PM20D1* promoter methylation	Prognostic	ADNI longitudinal cohort with ROSMAP brain confirmation	649 total in ADNI	Blood, brain tissue in replication	Not specified	Human	Whole-genome blood methylation microarray with longitudinal LME modeling	Wang et al., 2020
*PM20D1* DMR	Prognostic	ADNI progression EWAS	649 total in ADNI	Blood	Not specified	Human	Infinium MethylationEPIC	Wang et al., 2020
3-CpG blood DNA methylation	Prognostic	Betula longitudinal case-control with AIBL external replication	50 AD, 51 controls; external replication:146 AD, 324 controls	Blood	Not specified	Human	Illumina EPIC plus longitudinal mixed models/sPLS-DA	Schäfer Hackenhaar et al., 2023
15-ncRNA plasma signature	Prognostic	ACE nested case-control MCI cohort	192 participants	Plasma	Not specified	Human	Small RNA-seq with elastic net/Cox models	Gutierrez-Tordera et al., 2024
Mitochondrial D-loop methylation	Prognostic	8-years longitudinal peripheral blood cohort	75 participants	Blood	Not specified	Human	Longitudinal methylation analysis with linear mixed-effects modeling	Rizzo et al., 2026
AgeAccelGrim2 epigenetic clock	Prognostic	Women’s health initiative memory study	6,069 cognitively unimpaired women	Blood	Not specified	Human	DNAm epigenetic clock modeling	Nguyen et al., 2026

### 3.2 Therapeutic opportunities

Despite decades of investigation, therapeutic options with robust disease-modifying potential remain limited. Currently, the treatment of AD is primarily focused on symptomatic management with acetylcholinesterase inhibitors and *N*-methyl D-aspartate receptor antagonists, which induce only moderate and transitory improvements in cognitive and behavioral symptoms (Dineshbhai et al., 2026; Varinthra et al., 2026). However, the therapeutic landscape of AD has recently evolved with the introduction of disease-modifying anti-amyloid therapies. In particular, monoclonal antibodies, such as Lecanemab and Donanemab, have demonstrated a modest slowing of cognitive and functional decline in selected patients with early AD and confirmed amyloid pathology. Nevertheless, their use is currently restricted to specific patient populations and requires careful assessment of eligibility, potential benefits and risks, and appropriate monitoring (van Dyck et al., 2023; Rabinovici et al., 2025). The limited efficacy of currently available treatments underscores the need for novel therapeutic targets that may open new treatment strategies. In this context, epigenetic biomarkers are gaining increasing attention as promising therapeutic targets, in addition to their promising role as diagnostic and prognostic biomarkers (Jeremic et al., 2023).

Since 2018, several clinical trials have investigated therapeutic approaches targeting different layers of epigenetic regulation in AD, including repurposed drugs and novel epigenetic compounds (Jeremic et al., 2023). In this section, we report some examples of promising trials that have shown encouraging results. Notably, the ETHERAL-US trial is a Phase IIa clinical study (NCT03867253) conducted by Oryzon Genomics S.A. to evaluate the efficacy and safety of oral Vafidemstat (ORY-2001) in patients with mild-to-moderate AD (Oryzon Genomics, 2021). Vafidemstat is a brain-penetrant inhibitor of the epigenetic enzyme lysine-specific demethylase 1 (LSD1) which showed a favorable safety and tolerability profile. Topline data indicated that the drug significantly reduced the neuroinflammatory biomarker YKL-40, an astrocyte-secreted glycoprotein found in CSF and blood of AD patients (Oryzon Genomics, 2021). Following these results, the clinical development of Vafidemstat in AD has further expanded, focusing specifically on its potential to mitigate neuropsychiatric symptoms such as agitation and aggressiveness in more advanced stages of pathology. Specifically, the Phase IIa study named REIMAGINE-AD evaluated oral Vafidemstat in patients with moderate-to-severe AD presenting with agitation and aggression. The treatment was associated with significant improvements in several measures of agitation and aggression, with benefits maintained during longer-term treatment, although the study was small and open-label and therefore requires confirmation in larger controlled trials (Clinical Trials, 2026). Another approach involved Sulforaphane, a natural compound capable of modulating epigenetic pathways, which was investigated in patients with prodromal-to-mild AD in a randomized placebo-controlled trial (NCT04213391) (Second Affiliated Hospital, Zhejiang University, and School of Medicine, 2020; Gasparello et al., 2021). Gemfibrozil was also investigated for its ability to modulate miR pathways in predementia AD. The NCT02045056 trial, led by the University of Kentucky, evaluated 72 participants over 48 weeks to assess whether the drug could increase miRNA-107 levels. The trial demonstrated that Gemfibrozil is safe, brain-penetrant, and successfully modulates microRNA-107 levels, while showing positive trends in reducing toxic AD biomarkers and slowing brain atrophy (Walgrave et al., 2021). Overall, these studies illustrate the growing interest in targeting epigenetic pathways. While these innovative approaches are in their early stages, they could represent the next frontier of treatment and show great potential as ongoing clinical studies pave the way for promising results.

## 4 Conclusions, remarks and future directions

Alzheimer’s disease is an age-related neurodegenerative disorder characterized by progressive cognitive decline and memory loss, significantly impairing patients’ quality of life (Kamatham et al., 2024; Paniri et al., 2024; Tenchov et al., 2024). The progressive increase in life expectancy has led to an increase in the global prevalence of AD, generating social, economic, and healthcare burdens and representing one of the most pressing public health challenges of the twenty-first century (Gianfredi et al., 2025). The multifactorial nature of the disease results from the dynamic interplay between genetic susceptibility, aging-related molecular changes, environmental factors, and lifestyle-related exposures (Ahmadi et al., 2025; Mertaş and Boşgelmez, 2025). Despite substantial progress in clarifying AD pathophysiological mechanisms, the early diagnosis of at-risk individuals and the prediction of disease progression remain challenging issues. In this context, epigenetic mechanisms have emerged as key regulators bridging aging-associated biological changes and environmental influences on gene expression and cellular function, providing a novel perspective for gaining insight into AD susceptibility and progression (Villa and Combi, 2024; Kumari et al., 2025; Wang et al., 2025).

The evidence discussed in this review highlights the contribution of epigenetic dysregulation to key pathological processes involved in AD, including Aβ accumulation, tau pathology, neuroinflammation, synaptic dysfunction, oxidative stress, and mitochondrial impairment (Misrani et al., 2021; Ahmadi et al., 2025; D’Alessandro et al., 2025). Alterations in DNA methylation, histone modifications, ncRNAs (i.e., miRNAs, circRNAs, lncRNAs), mitochondrial epigenetics, and RNA methylation represent important molecular mechanisms through which epigenetic regulation may influence neuronal vulnerability and disease progression (Huang et al., 2020; Breen et al., 2023; Ding et al., 2023; Santana et al., 2023; Huang S. et al., 2025; Kashif and Said, 2025; Lukacsovich et al., 2025). In this context, aging represents a central biological driver, as it is associated with progressive epigenomic remodeling, including changes in DNA methylation patterns, chromatin accessibility, and epigenetic clock profiles (Thrush et al., 2022; Liu Y. et al., 2024; Zhao et al., 2025). Age-related alterations may contribute to the increased susceptibility of the aging brain to neurodegenerative processes (Liu Y. et al., 2024).

In addition to their mechanistic significance in AD pathogenesis, epigenetic alterations represent a promising and rapidly expanding class of emerging biomarkers in AD, providing valuable information for early diagnosis, risk assessment, and disease monitoring (Wisman et al., 2024). Unlike genetic variants, epigenetic modifications are dynamic and potentially reversible. These characteristics make them particularly attractive as candidate biomarkers capable of capturing the complex and heterogeneous nature of AD and as therapeutic targets potentially modifiable to restore physiological gene expression patterns (Wisman et al., 2024; Tisato et al., 2025). Nevertheless, several challenges currently limit the clinical application of epigenetic biomarkers (Wisman et al., 2024). These include the need for validation in large and well-characterized longitudinal cohorts, the standardization of analytical pipelines and data interpretation, and a deeper understanding of whether specific epigenetic alterations represent causal contributors or consequences of disease neurodegeneration, and more broadly, of disease pathogenesis (Wisman et al., 2024; Chu et al., 2026). An additional challenge is represented by cellular heterogeneity, particularly in studies based on bulk brain tissue, where epigenetic alterations may reflect changes in cellular composition rather than cell-intrinsic epigenetic remodeling. Recent advances in single-cell and single-nucleus multiomic approaches are enabling the characterization of cell-type- and region-specific epigenomic alterations in AD, providing a more precise understanding of disease-associated molecular changes and cellular vulnerability (Liu Z. et al., 2025; Shi et al., 2025). Furthermore, emerging spatial epigenomics techniques are beginning to map these modifications directly within their histological context (Liu Z. et al., 2025). In parallel, computational deconvolution approaches applied to bulk methylomic and chromatin-accessibility data may help resolve cell-type-specific epigenetic signatures in large cohorts (Yap et al., 2024). Moreover, the accessibility of brain tissues and brain-derived biofluids (i.e., CSF) represents a major limitation for clinical translation (Villa and Combi, 2024). Therefore, future research should increasingly focus on identifying robust epigenetic signatures detectable in peripheral biological samples, primarily blood, with the aim of developing minimally invasive liquid biopsy-based diagnostic/prognostic approaches (Apostolov et al., 2026). Current research efforts are moving toward the identification and validation of blood-derived epigenetic biomarkers, including circulating cell-free (cf) genomic and mtDNA methylation, which could represent an important step toward improving early AD diagnosis, patient stratification, and disease progression assessment (Bahado-Singh et al., 2022; Zhen et al., 2025; Apostolov et al., 2026). For example, plasma cfDNA methylation profiling has identified disease-associated signatures in AD, including *CTBP1* promoter hypermethylation (Dong et al., 2025), while neuron-derived cfDNA methylation signatures have shown potential for distinguishing AD from controls and predicting MCI-to-AD progression (Pollard et al., 2025). Similarly, mtDNA methylation studies have reported reduced D-loop methylation in peripheral blood of AD patients, and more recent longitudinal work suggests that mtDNA methylation patterns may help predict cognitive decline in MCI (Stoccoro et al., 2022; Gascón-Bayarri et al., 2025; Rizzo et al., 2026).

Furthermore, the integration of epigenomic data with emerging artificial intelligence and machine learning approaches in clinical practice represents another promising avenue for personalized management of AD (Ren et al., 2025; D’Aversa et al., 2026). The increasing availability of high-dimensional multi-omic molecular datasets, that could integrate DNA/RNA methylation signatures, ncRNA expression patterns, clinical parameters, and neuroimaging data, provides an opportunity to develop computational models capable of identifying complex epigenetic patterns associated with AD onset and progression across individuals, supporting earlier intervention strategies (Ren et al., 2025).

In conclusion, epigenetics represents an emerging and dynamic field that provides novel insights into the molecular complexity of AD while opening new opportunities for biomarker discovery. Although further validation is required before clinical implementation, epigenetic biomarkers supported by computational models hold significant potential for a more accurate, minimally invasive, and personalized approach for AD diagnosis and prognosis. At the same time, the identification of disease-associated epigenetic alterations may provide novel therapeutic targets and support the development of innovative strategies aimed at modulating dysregulated epigenetic mechanisms and restoring physiological gene expression patterns.
